# Spatio-temporal inequalities in vulvar cancer mortality in Spain, 1999–2023: a nationwide Bayesian analysis

**DOI:** 10.1007/s12094-026-04408-3

**Published:** 2026-05-25

**Authors:** Lucía Cayuela, José-Juan Pereyra-Rodríguez, Julián Librero, María Pastor-Valero, Aurelio Cayuela

**Affiliations:** 1 Department of Internal Medicine, Hospital Severo Ochoa, Leganés, Spain; 2 Department of Medicine, University of Seville, Seville, Spain; 3 Department of Dermatology, Virgen del Rocío University Hospital, Avda. Manuel Siurot s/n, 41013 Seville, Spain; 4 Red de Investigación en Servicios de Salud en Enfermedades Crónicas (REDISSEC), Navarrabiomed-Fundación Miguel Servet, Pamplona, Spain; 5 Departamento de Salud Pública, Historia de la Ciencia y Ginecología, Facultad de Medicina, Universidad Miguel Hernández, Sant Joan d’Alacant, Alicante, Spain; 6 CIBER in Epidemiology and Public Health (CIBERESP), Madrid, Spain; 7 Programa de Pós Graduação em Saúde Coletiva, Faculdade de Medicina da Universidade de São Paulo, São Paulo, SP, Brazil; 8 Unit of Public Health, Prevention and Health Promotion, South Seville Health Management Area, Seville, Spain

**Keywords:** Vulvar neoplasms, Mortality, Spain, Bayesian analysis, Spatio-temporal analysis, Small-area analysis

## Abstract

**Objective:**

To conduct the first nationwide spatio-temporal analysis of vulvar cancer mortality in Spain, identifying geographical inequalities and temporal trends over 25 years.

**Methods:**

A population-based ecological study analyzed mortality data (1999–2023) across 52 Spanish provinces and autonomous cities. Expected deaths were calculated via indirect standardization. Hierarchical Bayesian spatio-temporal models were implemented using Integrated Nested Laplace Approximation (INLA).

**Results:**

Spatial heterogeneity accounted for 68.8% of the total posterior variance, whereas the national temporal trend explained only 8.2%. Nationally, mortality risk showed a modest downward trend, with the RR decreasing from 1.054 (95% CrI 0.981–1.133) in 1999 to 0.954 (95% CrI 0.890–1.022) in 2023. However, areas with a high posterior probability (PP(RR > 1) > 0.95) of excess mortality were consistently identified in southwestern Spain (Badajoz RR 1.25, Cádiz 1.21, Huelva 1.20, Sevilla 1.18) and Lugo (RR 1.16), while lower risks were observed in Madrid and Barcelona (RR 0.85).

**Conclusions:**

Vulvar cancer mortality in Spain shows persistent geographical disparities rather than a uniform national trend. These ecological findings do not allow causal interpretation, but they may reflect underlying contextual factors, such as socioeconomic inequalities, heterogeneous access to early diagnosis, variability in access to specialized gynecologic oncology services, and differences in the detection and management of precursor conditions. These results highlight the need for further research incorporating incidence, clinical stage, and healthcare access data, as well as public health strategies aimed at ensuring equitable access to multidisciplinary care and strengthening coordinated early detection pathways across regions.

**Supplementary Information:**

The online version contains supplementary material available at https://doi.org/10.1007/s12094-026-04408-3.

## Introduction

Vulvar cancer is an uncommon yet clinically significant gynecologic malignancy. It is increasingly diagnosed in many high-income countries and continues to cause substantial mortality worldwide [1, 2]. According to GLOBOCAN 2022, vulvar cancer accounted for 47,336 new cases and 18,579 deaths globally, with age-standardized incidence and mortality rates of 0.83 and 0.30 per 100,000, respectively. While Europe bears a significant share of the burden, the disease predominantly affects postmenopausal women, with diagnosis typically occurring in the late 60 s or early 70 s [3, 4].

Recent population-based studies show sustained increases in incidence, particularly among younger women, while mortality and survival trends remain heterogeneous across countries and age groups [1–9]. This variation likely stems from differences in risk factor prevalence, healthcare access, and diagnostic pathways. Pathogenically, two distinct routes are recognized: an HPV-associated pathway, more common in younger cohorts, and an HPV-independent pathway, typically linked to chronic inflammatory dermatoses such as lichen sclerosus in older women [9–11]. These pathways explain the shifting age patterns of the disease—with HPV-associated tumors contributing to rising incidence in younger women, while non-HPV-related disease appears comparatively stable—and highlight the long-term potential of HPV vaccination to reduce future disease burden [9–13].

In Spain, evidence is limited and largely derived from regional registries, suggesting modest increases in incidence and persistent mortality despite improvements in survival [14, 15]. Furthermore, national geographical inequalities remain poorly characterized, and no nationwide spatio-temporal analysis of vulvar cancer mortality has been performed. This is a critical gap, as Spain’s marked demographic diversity and regional variation in healthcare organization may significantly influence mortality outcomes.

Small-area spatio-temporal modeling offers a robust way to study rare cancers by borrowing strength across neighboring areas and time periods, thereby producing more stable estimates than crude rates alone [16, 17]. Bayesian hierarchical approaches using Integrated Nested Laplace Approximation (INLA) are particularly well suited to this purpose and have been successfully applied to cancer mortality mapping in Spain and other settings [18]. Against this background, the present study provides the first nationwide spatio-temporal analysis of vulvar cancer mortality in Spain from 1999 to 2023, estimating provincial relative risks while accounting for spatial and temporal dependence to identify previously unrecognized geographical inequalities that may inform targeted healthcare planning and resource allocation.

## Methods

### Study design and data sources

We conducted a nationwide, population‑based ecological study to examine the spatio‑temporal distribution of vulvar cancer mortality in Spain between 1999 and 2023. The analysis covered the 50 Spanish provinces and the two autonomous cities of Ceuta and Melilla, yielding a total of 52 geographical units.

Mortality data were obtained from the National Institute of Statistics, and cases were identified using the International Classification of Diseases, 10th Revision (ICD-10) code C51. Annual population counts stratified by province, year, and five-year age groups were retrieved from the same source. Given the rarity of vulvar cancer and the marked heterogeneity in the age structure of the Spanish population, the expected number of deaths *E*_*it*​_ for each province–year combination was estimated by indirect standardization. National age-specific mortality rates for the entire study period were used as the reference population.

### Statistical analysis and spatio-temporal modeling

Because vulvar cancer is a rare malignancy, mortality rates can be unstable, particularly in provinces with small populations. To obtain more reliable estimates of geographical inequalities, we applied Bayesian hierarchical spatio‑temporal models that “borrow strength” across neighboring provinces and adjacent years.

Let *y*_it_ denote the observed number of deaths in province *i* during year *t*. Mortality counts were assumed to follow a Poisson distribution: y_it_ ~ Poisson (E_it_ × θ_it_), where E_it_ is the expected number of deaths and *θ*_it_ is the relative risk (RR). The log‑relative risk was modeled as: log(θ_it_) = α + S_i_ + T_t_ + ST_it,_ where *α* represents the overall intercept, *S*_i_ the spatial component, *T*_t_ the temporal component, and ST_it_ the space–time interaction term, which captures province-specific temporal deviations from the national trend.

A comprehensive model selection strategy was implemented to ensure robustness of the final specification. Candidate models were constructed by systematically combining alternative assumptions regarding: (i) four spatial structures (BYM, BYM2, iCAR, and Leroux), (ii) two temporal structures (first- and second-order random walks, RW1 and RW2), (iii) four interaction formulations (Types I–IV and additive specifications), and (iv) the inclusion or exclusion of Penalized Complexity (PC) priors. This factorial design resulted in 80 candidate models, allowing a thorough assessment of sensitivity to structural and prior assumptions [17].

Model performance was evaluated using Deviance Information Criterion (DIC) and Watanabe–Akaike Information Criterion (WAIC), as summarized in Supplementary Table S1. The comparison showed a high degree of consistency across specifications, with only marginal differences among the best-performing models, indicating that inference is robust to reasonable changes in model structure and prior choices.

Posterior distributions of the relative risk were used to identify provinces and periods with excess mortality. For each province-year combination, the posterior probability that the relative risk exceeded 1, PP (RR_it_ > 1), was calculated. Posterior probabilities < 0.20 were interpreted as low evidence of excess risk, values between 0.20 and 0.80 as moderate evidence, values > 0.80 as strong evidence, and values ≥ 0.95 as very strong evidence.

The spatial distribution and temporal evolution of mortality risk were subsequently represented using choropleth maps and temporal trend plots to identify high-risk areas and support targeted public health interventions.

All analyses were conducted using RStudio version 2025.05.1 + 513 and the R-INLA package version 25.06.07.

## Results

During 1999–2023, vulvar cancer caused 7543 deaths among Spanish women, with a median of 145 deaths per province (IQR 104–217; range 6–816). The highest crude numbers occurred in Madrid (816 deaths), Barcelona (779), A Coruña (263), Valencia (347), and Sevilla (315), reflecting both population size and underlying risk.

Among the 80 Bayesian spatio‑temporal models evaluated, the best‑fitting model for vulvar cancer mortality in women incorporated a Leroux spatial structure, a first‑order random walk (RW1) for the temporal effect, and a Type II space–time interaction according to the Knorr–Held classification, without Penalized Complexity (PC) priors. This specification yielded the lowest Deviance Information Criterion (DIC = 5444.48) and Widely Applicable Information Criterion (WAIC = 5442.37). Models with intrinsic conditional autoregressive and Besag–York–Mollié 2 spatial structures and identical temporal and interaction components produced very similar DIC and WAIC values, indicating robustness to the choice of spatial parameterization.

Sensitivity analysis comparing models with and without PC priors showed that PC priors induced strong shrinkage of the space–time interaction term, reducing its posterior variance and the number of provinces identified as high‑risk. The model without PC priors achieved better fit in terms of information criteria and posterior predictive checks and was therefore retained as the final model.

Variance decomposition of the final model showed that spatial heterogeneity accounted for 68.8% of the total posterior variance, the space–time interaction for 23.0%, and the temporal component for only 8.2%. These findings indicate that geographical differences between provinces were considerably more important than the overall national temporal trend, and that a substantial proportion of variability arose from province‑specific changes over time.

**Fig. 1:**
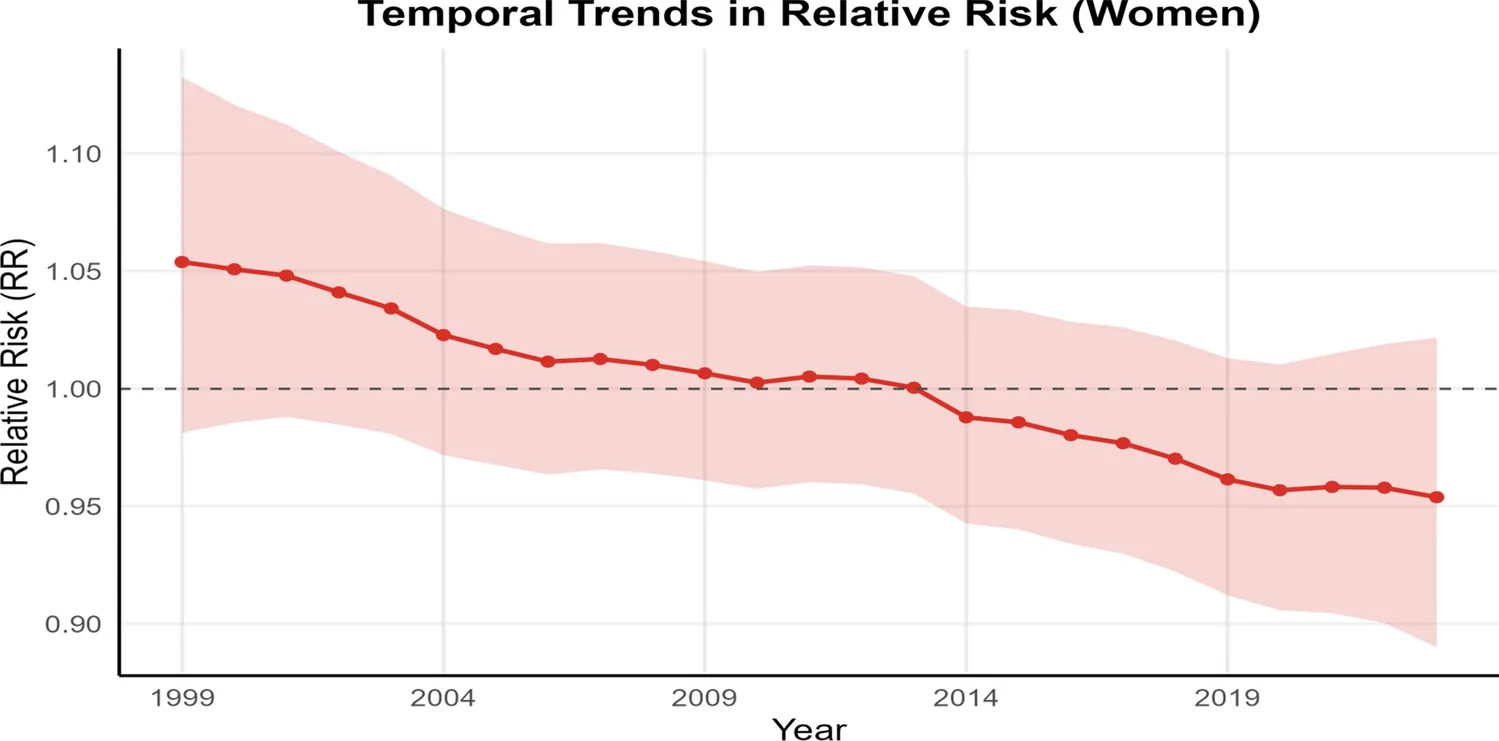
National temporal trend in vulvar cancer mortality risk among women in Spain, 1999–2023. The solid red line represents the posterior mean of the Relative Risk (RR), with the light red shaded area indicating the 95% credible interval. The horizontal dashed line at RR = 1.00 serves as the reference baseline, representing the average risk over the entire study period. Data points (circles) indicate annual estimates, showing a progressive decline in mortality risk from 1999 to 2023

Figure 1 displays the national temporal effect. We observed a modest downward trend in the relative risk (RR) over the study period, from 1.054 (95% CrI 0.981–1.133) in 1999 to 0.954 (95% CrI 0.890–1.022) in 2023. As 95% CrIs included unity throughout, these results do not support a definitive decline. However, the posterior probability of excess risk (RR > 1) decreased markedly from 0.92 in 1999 to 0.09 in 2023, suggesting a shift toward a lower-risk scenario in recent years.

**Fig. 2:**
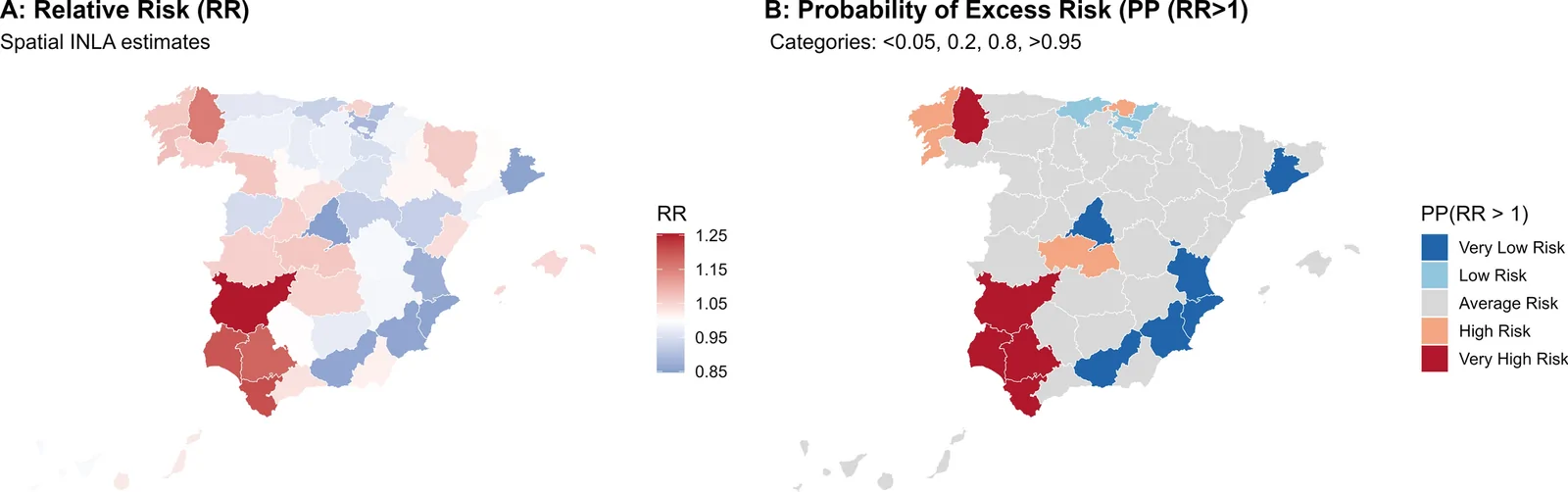
Smoothed overall relative risk (RR) and posterior probability of excess mortality for vulvar cancer by province, 1999–2023. Left Map: Shows the distribution of relative risk by sextiles. Right Map: Displays the posterior probability PP (RR > 1)

Smoothed overall relative risks (RR) for the full study period (1999–2023) revealed substantial geographical heterogeneity across the 52 spatial units (Fig. 2). Five provinces showed strong evidence of excess mortality, defined as a posterior probability of relative risk greater than 1 of at least 0.95. The highest risk was observed in Badajoz (relative risk 1.25, 95% CrI 1.10–1.43), followed by Cádiz (1.21, 95% CrI 1.07–1.36), Huelva (1.20, 95% CrI 1.04–1.39), Sevilla (1.18, 95% CrI 1.07–1.31), and Lugo (1.16, 95% CrI 1.00–1.34).

Conversely, six provinces showed significantly lower mortality risk, with a posterior probability of relative risk greater than 1 of 0.05 or lower. These provinces were Madrid (relative risk 0.85, 95% CrI 0.79–0.91), Barcelona (0.85, 95% CrI 0.80–0.92), Valencia (0.87, 95% CrI 0.80–0.96), Alicante (0.86, 95% CrI 0.76–0.96), Murcia (0.86, 95% CrI 0.75–0.98), and Granada (0.86, 95% CrI 0.75–0.99). The remaining provinces presented relative risks close to the national average and lacked strong statistical evidence of either excess or reduced mortality.

The space–time interaction indicated that these geographical patterns remained remarkably stable over the 25 year period. Provinces with persistently elevated mortality risk clustered in southern and western Spain, particularly in Andalusia and Extremadura, whereas provinces with consistently lower mortality risk were concentrated in the northeast and along the Mediterranean coast. Large urban areas, including Madrid and Barcelona, maintained lower‑than‑expected mortality throughout the study period, with only modest temporal shifts within individual provinces.

**Fig. 3:**
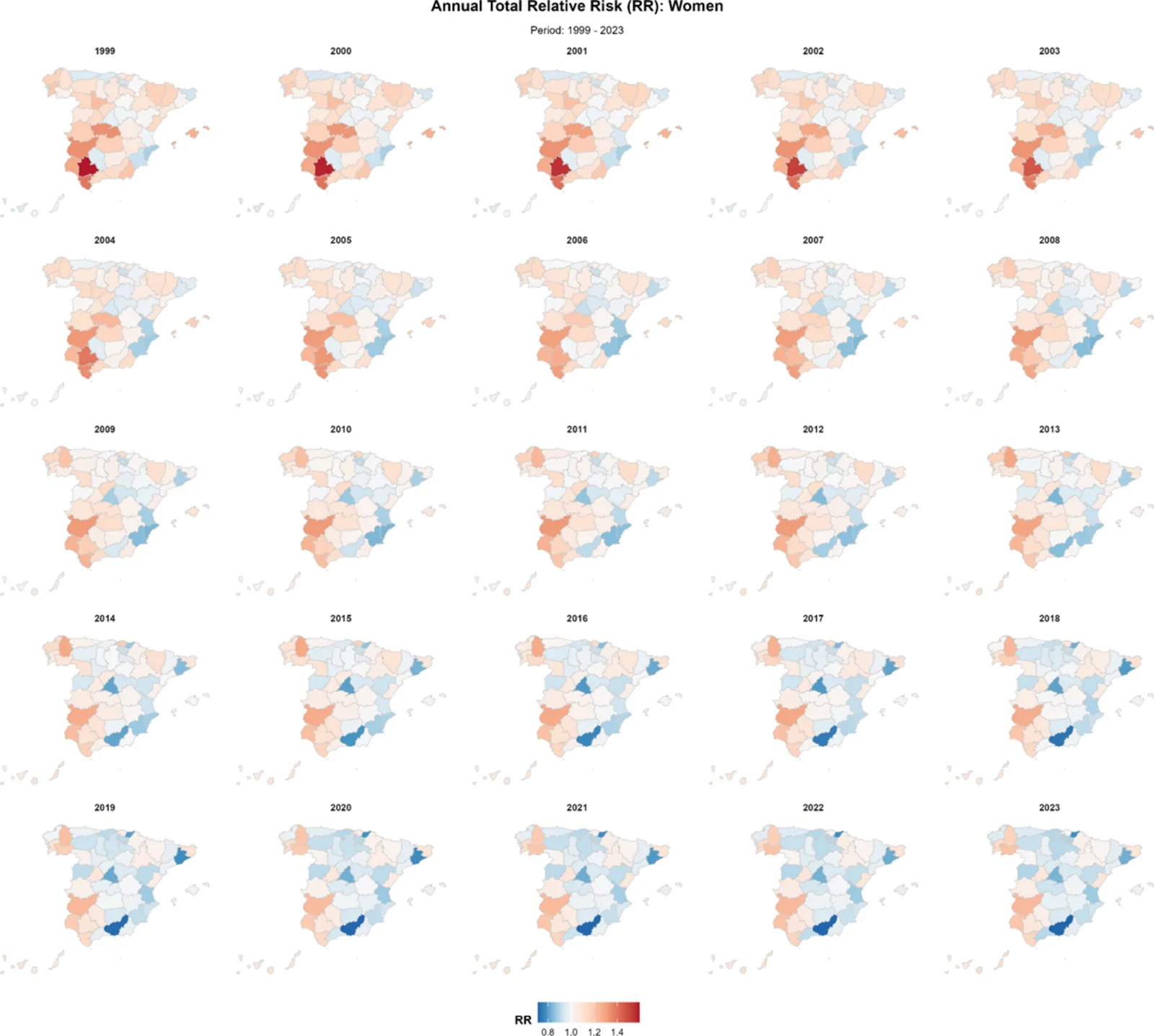
Spatiotemporal evolution of relative risk (RR) for vulvar cancer mortality in Spain (1999–2023). Annual maps represent the posterior mean of the provincial relative risk. Red areas indicate an RR > 1.0 (increased risk), and blue areas indicate an RR < 1.0 (decreased risk) compared to the national average

**Fig. 4:**
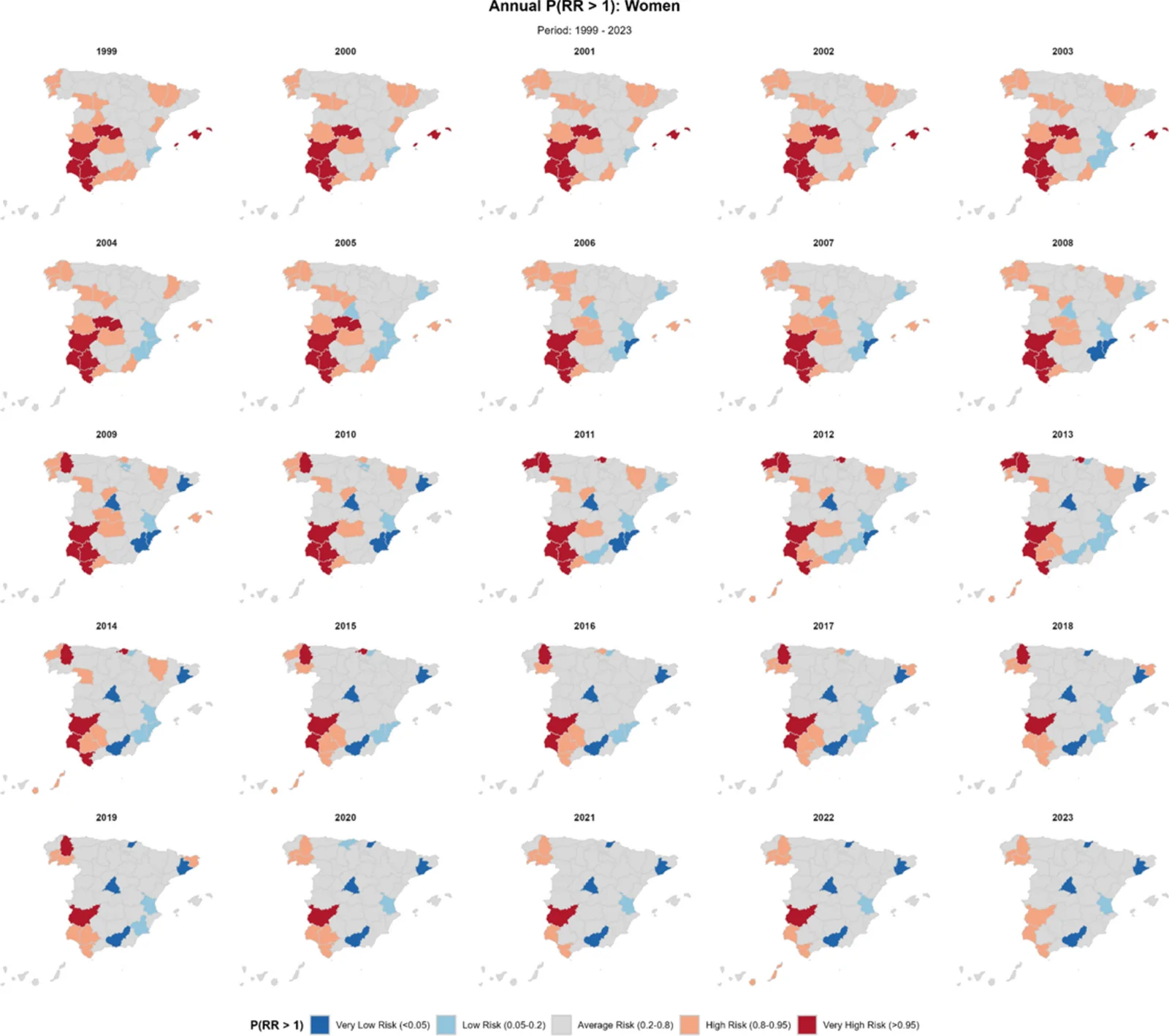
Posterior probability of increased mortality risk (PP (RR > 1)) by province and year. The categorical scale identifies areas with a very high probability of excess risk (dark red, > 0.95) and areas with a very low probability (dark blue, < 0.05)

The annual evolution of provincial relative risk and posterior probability of excess mortality (Figs. 3–4) confirmed that geographical patterns remained remarkably stable over the 25 years. High‑risk provinces clustered consistently in southwestern Spain (particularly Andalusia and Extremadura), while low‑risk provinces concentrated along the Mediterranean coast and northeast.

## Discussion

This study provides the first nationwide spatio-temporal analysis of vulvar cancer mortality in Spain over a 25 year period (1999–2023). Bayesian hierarchical modeling identified three key patterns: a modest downward trend in national mortality risk; a concentration of excess mortality with high posterior probability in southwestern Spain (Badajoz, Cádiz, Huelva, Sevilla) and Lugo; and a predominance of spatial over temporal variation (68.8%).

The modest decline observed is broadly consistent with patterns reported in other high-income European countries, where mortality has remained stable or decreased slightly despite stable or increasing incidence [7, 19]. Several clinical advances may plausibly contribute to this trend in Spain. Over the past two decades, improvements in surgical staging, adoption of sentinel lymph node biopsy, enhanced pathologic classification, centralization to high-volume centers, and expanded multidisciplinary care, including adjuvant radiotherapy, have likely enhanced outcomes [20]. Although this study cannot directly assess their impact due to the absence of individual-level treatment data, the temporal pattern is consistent with these developments.

Nevertheless, the national decline remains modest. Relative risk decreased only slightly over the study period, and the temporal component explained a small fraction of overall variability. Given that vulvar cancer is potentially detectable at an early stage, the limited magnitude of improvement may suggest that a substantial proportion of women continue to be diagnosed at advanced disease stages or experience barriers along the diagnostic and treatment pathway although this cannot be directly evaluated with mortality data alone.

Several interacting mechanisms may plausibly contribute to this pattern though these remain hypotheses given the ecological design. Diagnostic delays are common in vulvar cancer due to non-specific early symptoms overlooked by patients and primary care providers. Limited clinician awareness and patient stigma further exacerbate delays [11, 21]. Older women, the highest-risk group, are particularly vulnerable; the AGO-CaRE-1 study found those ≥ 70 years presented with larger tumors, advanced stage, and nodal involvement [22].

The persistence of excess mortality despite vulvar cancer’s theoretical early detectability likely reflects absent systematic screening. Unlike cervical cancer, no population-based vulvar screening exists; diagnosis relies on symptom recognition and referral. This disproportionately affects older women [23], with EUROCARE-5 linking poorer European survival to late detection and access barriers [6]. In our study, these factors should be interpreted as potential contributors rather than demonstrated explanations.

Unlike temporal trends, spatial factors explained about 70% of the posterior variance, indicating that vulvar cancer mortality in Spain is driven more by persistent local disparities than by national trends. This aligns with previous studies of other cancers in Spain [24], which highlight the influence of geographical context on outcomes.

Strong evidence of excess mortality was concentrated in Extremadura and western Andalusia, particularly Badajoz, Cádiz, Huelva, and Sevilla, whereas the lowest risks were observed in Madrid, Barcelona, and several eastern Mediterranean provinces. The persistence of these patterns over time supports the presence of stable spatial inequalities rather than random variation.

Several mechanisms may plausibly underlie the excess mortality identified in southwestern Spain. First, socioeconomic deprivation is a potential contributor, as this region has historically shown less favorable socioeconomic indicators [25], which are often associated with delayed diagnosis and reduced access to specialized care [26]. Second, differences in the availability of and access to specialized oncologic services may influence outcomes. Management of vulvar cancer requires multidisciplinary expertise, and geographical barriers to referral centers could affect the timeliness of diagnosis and treatment. These findings suggest that strengthening referral pathways and ensuring equitable access to specialized gynecologic oncology services, potentially through further centralization of care, is a priority for higher-risk regions. Third, variability in coordination between gynecology and dermatology for precursor conditions may influence early detection. Third, the dual etiology of vulvar cancer may also partly explain the observed heterogeneity [11]. The HPV-related pathway, which predominates in younger women, may vary regionally according to differences in HPV prevalence, smoking behavior, and sexual practices [9]. Conversely, the HPV-independent pathway, linked to lichen sclerosus and other chronic inflammatory dermatoses in older women [27], may show distinct regional variations. Relatedly, variability in coordination between gynecology and dermatology for these precursor conditions could influence early detection. The low mortality observed in Madrid and Barcelona aligns with the concentration of specialized centers in these areas [28] though the absence of nationwide data on HPV status or histology precludes direct assessment of these hypotheses [29] and the ecological design prevents causal inference.

The high-risk cluster identified in Lugo may reflect contextual factors, such as population aging, rurality, and healthcare accessibility. Although age standardization was applied, residual contextual influences cannot be excluded.

These findings have specific implications for clinical practice and health system organization in high-risk provinces. First, enhanced referral pathways between dermatology and gynecology are needed to ensure timely recognition and management of precursor conditions such as lichen sclerosus, particularly in southwestern Spain. Second, greater centralization of vulvar cancer care in high-volume gynecologic oncology centers serving Extremadura and western Andalusia could improve outcomes, following the model observed in Madrid and Barcelona. Third, targeted clinician training programs on vulvar disease symptoms and early diagnostic strategies should be prioritized in high-risk regions to reduce diagnostic delays.

Our results also have implications for future prevention strategies. Spain introduced prophylactic HPV vaccination for adolescent girls in 2008, and vaccine coverage is now high across most regions. Given the long latency and rarity of vulvar cancer, a measurable impact on invasive cancer mortality is unlikely to be detectable for several decades; however, reductions in high-grade vulvar intraepithelial neoplasia have already been documented in other countries with established vaccination programs. The modest downward mortality trend observed during the study period therefore more plausibly reflects improvements in clinical care than any vaccination effect. Looking forward, sustained high vaccine coverage could contribute to reducing geographical inequalities in vulvar cancer mortality if uptake remains equitable across all provinces.

The present study has several notable strengths. It covers the entire Spanish population over a 25-year period and applies advanced Bayesian spatio-temporal methods specifically designed for rare disease analyses. By borrowing information across neighboring provinces and adjacent years, the models produce stable estimates even in areas with few observed deaths. The consistency of findings across alternative spatial structures further supports the robustness of the results.

Several limitations must nonetheless be acknowledged. First, the ecological design precludes causal inference at the individual level and is susceptible to ecological fallacy. Second, mortality data contain no information on tumor stage, histological subtype, HPV status, socioeconomic position, or treatment access—all factors that may substantially influence survival. Third, despite Bayesian smoothing, residual uncertainty remains in provinces with very few deaths. Finally, because the study examined mortality rather than incidence, it cannot distinguish whether observed geographical differences arise from variation in disease occurrence, survival, or both.

## Conclusion

In conclusion, vulvar cancer mortality in Spain exhibited a modest downward trend from 1999 to 2023, yet marked geographical inequalities persisted throughout the study period. Southwestern provinces showed a high posterior probability of excess mortality, while lower risks were concentrated in large urban and Mediterranean areas. These disparities plausibly reflect unequal access to specialized diagnosis and treatment, variations in the management of precursor conditions, and potential socioeconomic differences. To translate these findings into clinical practice, public health efforts should focus on optimizing referral pathways from primary care, improving coordination between gynecology and dermatology, and ensuring the centralization of specialized oncologic care to guarantee equitable outcomes for women regardless of their province of residence.

## Supplementary Information

Below is the link to the electronic supplementary material.

Supplementary file1 (DOCX 24 KB)

Supplementary file2 (DOCX 25 KB)

Supplementary file3 (DOCX 20 KB)

## Data Availability

The data for this study are publicly available through the https://www.ine.es. All analyses were conducted using RStudio version 2025.05.1 + 513 and the R-INLA package version 25.06.07.
