# Supplementary material for: Spatio-temporal inequalities in vulvar cancer mortality in Spain, 1999–2023: a nationwide Bayesian analysis
Source: Clin Transl Oncol. 2026 May 25;28(11):5255–62. doi: 10.1007/s12094-026-04408-3 (PMC13694372; doi:10.1007/s12094-026-04408-3)
Supplement: Supplementary file 1 [file 12094_2026_4408_MOESM1_ESM.docx]

**Table S1. Model Selection and Sensitivity Analysis Comparison**

Goodness-of-fit statistics (DIC and WAIC) for the full candidate model space. The table evaluates combinations of intrinsic and Leroux spatial priors, first- and second-order random walks (RW) for time, and four types of space-time interactions. Comparative results for models with and without Penalized Complexity (PC) priors are provided as a sensitivity analysis, confirming that inference is robust to prior specification with no evidence of over-shrinkage.

| **Rank** | **Spatial** | **Temporal** | **Type** | **PC priors** | **DIC** | **WAIC** |
| --- | --- | --- | --- | --- | --- | --- |
| **1** | **Leroux** | **RW1** | **TypeII** | **No** | **5444.48** | **5442.37** |
| **2** | **iCAR** | **RW1** | **TypeII** | **Yes** | **5444.67** | **5442.36** |
| **3** | **BYM2** | **RW1** | **TypeII** | **Yes** | **5444.68** | **5442.13** |
| **4** | **BYM2** | **RW1** | **TypeII** | **No** | **5444.84** | **5441.72** |
| **5** | **iCAR** | **RW1** | **TypeII** | **No** | **5445.30** | **5442.77** |
| **6** | **BYM** | **RW1** | **TypeII** | **No** | **5445.85** | **5443.29** |
| **7** | **Leroux** | **RW1** | **TypeII** | **Yes** | **5445.86** | **5443.33** |
| **8** | **BYM** | **RW1** | **TypeII** | **Yes** | **5446.97** | **5444.60** |
| **9** | **BYM2** | **RW1** | **TypeIV** | **Yes** | **5452.17** | **5450.86** |
| **10** | **BYM** | **RW1** | **TypeIV** | **Yes** | **5452.99** | **5452.18** |
| **11** | **BYM2** | **RW1** | **TypeIV** | **No** | **5453.32** | **5452.61** |
| **12** | **Leroux** | **RW1** | **TypeIV** | **No** | **5453.63** | **5452.87** |
| **13** | **BYM** | **RW1** | **TypeIV** | **No** | **5454.06** | **5453.25** |
| **14** | **Leroux** | **RW1** | **TypeIV** | **Yes** | **5454.37** | **5454.12** |
| **15** | **iCAR** | **RW1** | **TypeIV** | **No** | **5454.74** | **5453.95** |
| **16** | **iCAR** | **RW1** | **TypeIV** | **Yes** | **5456.77** | **5456.75** |
| **17** | **BYM** | **RW2** | **TypeI** | **Yes** | **5461.19** | **5464.00** |
| **18** | **Leroux** | **RW2** | **TypeI** | **Yes** | **5462.47** | **5463.75** |
| **19** | **BYM2** | **RW2** | **TypeI** | **Yes** | **5462.52** | **5463.78** |
| **20** | **Leroux** | **RW2** | **TypeIII** | **No** | **5462.63** | **5463.87** |
| **21** | **Leroux** | **RW2** | **TypeIII** | **Yes** | **5462.75** | **5463.90** |
| **22** | **Leroux** | **RW2** | **TypeI** | **No** | **5462.80** | **5463.93** |
| **23** | **BYM2** | **RW2** | **TypeI** | **No** | **5462.80** | **5463.88** |
| **24** | **BYM2** | **RW2** | **Additive** | **Yes** | **5463.21** | **5464.50** |
| **25** | **Leroux** | **RW2** | **Additive** | **Yes** | **5463.21** | **5464.49** |
| **26** | **BYM2** | **RW2** | **Additive** | **No** | **5463.30** | **5464.60** |
| **27** | **BYM** | **RW2** | **Additive** | **Yes** | **5463.34** | **5464.60** |
| **28** | **Leroux** | **RW2** | **Additive** | **No** | **5463.36** | **5464.66** |
| **29** | **BYM** | **RW2** | **Additive** | **No** | **5463.50** | **5464.77** |
| **30** | **iCAR** | **RW2** | **Additive** | **Yes** | **5463.76** | **5464.87** |
| **31** | **iCAR** | **RW2** | **Additive** | **No** | **5463.96** | **5465.08** |
| **32** | **BYM** | **RW1** | **TypeIII** | **Yes** | **5464.06** | **5465.38** |
| **33** | **iCAR** | **RW1** | **TypeI** | **Yes** | **5464.22** | **5466.56** |
| **34** | **BYM2** | **RW2** | **TypeIII** | **No** | **5464.25** | **5465.40** |
| **35** | **BYM2** | **RW1** | **TypeIII** | **Yes** | **5464.44** | **5465.72** |
| **36** | **Leroux** | **RW1** | **TypeI** | **Yes** | **5464.69** | **5466.01** |
| **37** | **BYM2** | **RW1** | **TypeI** | **Yes** | **5464.79** | **5466.06** |
| **38** | **iCAR** | **RW2** | **TypeI** | **Yes** | **5465.02** | **5464.36** |
| **39** | **BYM** | **RW2** | **TypeI** | **No** | **5465.10** | **5463.97** |
| **40** | **Leroux** | **RW1** | **TypeI** | **No** | **5465.12** | **5466.17** |
| **41** | **BYM** | **RW1** | **TypeI** | **Yes** | **5465.14** | **5466.39** |
| **42** | **iCAR** | **RW2** | **TypeIII** | **Yes** | **5465.18** | **5466.33** |
| **43** | **BYM2** | **RW1** | **TypeI** | **No** | **5465.18** | **5466.15** |
| **44** | **BYM2** | **RW1** | **Additive** | **Yes** | **5465.32** | **5466.76** |
| **45** | **Leroux** | **RW1** | **Additive** | **Yes** | **5465.33** | **5466.78** |
| **46** | **BYM2** | **RW1** | **Additive** | **No** | **5465.35** | **5466.78** |
| **47** | **iCAR** | **RW2** | **TypeI** | **No** | **5465.38** | **5464.49** |
| **48** | **Leroux** | **RW1** | **Additive** | **No** | **5465.44** | **5466.89** |
| **49** | **BYM** | **RW1** | **Additive** | **Yes** | **5465.49** | **5466.91** |
| **50** | **iCAR** | **RW2** | **TypeIII** | **No** | **5465.61** | **5467.24** |
| **51** | **BYM** | **RW1** | **Additive** | **No** | **5465.61** | **5467.04** |
| **52** | **BYM** | **RW2** | **TypeIII** | **Yes** | **5465.62** | **5467.07** |
| **53** | **Leroux** | **RW1** | **TypeIII** | **No** | **5465.75** | **5467.10** |
| **54** | **BYM** | **RW1** | **TypeIII** | **No** | **5465.79** | **5467.39** |
| **55** | **iCAR** | **RW1** | **Additive** | **Yes** | **5465.84** | **5467.12** |
| **56** | **BYM** | **RW2** | **TypeIII** | **No** | **5465.86** | **5467.12** |
| **57** | **iCAR** | **RW1** | **TypeIII** | **Yes** | **5465.93** | **5467.42** |
| **58** | **iCAR** | **RW1** | **Additive** | **No** | **5465.97** | **5467.26** |
| **59** | **BYM2** | **RW1** | **TypeIII** | **No** | **5466.26** | **5467.39** |
| **60** | **BYM** | **RW1** | **TypeI** | **No** | **5466.33** | **5466.40** |
| **61** | **BYM2** | **RW2** | **TypeIII** | **Yes** | **5466.44** | **5467.65** |
| **62** | **iCAR** | **RW1** | **TypeIII** | **No** | **5466.83** | **5468.28** |
| **63** | **iCAR** | **RW1** | **TypeI** | **No** | **5467.50** | **5466.76** |
| **64** | **Leroux** | **RW1** | **TypeIII** | **Yes** | **5469.52** | **5470.85** |
| **65** | **BYM** | **RW2** | **TypeII** | **Yes** | **5481.70** | **5477.47** |
| **66** | **Leroux** | **RW2** | **TypeIV** | **No** | **5482.19** | **5479.00** |
| **67** | **Leroux** | **RW2** | **TypeII** | **No** | **5482.43** | **5477.86** |
| **68** | **BYM2** | **RW2** | **TypeII** | **Yes** | **5482.47** | **5478.20** |
| **69** | **BYM** | **RW2** | **TypeII** | **No** | **5482.51** | **5477.93** |
| **70** | **iCAR** | **RW2** | **TypeII** | **No** | **5482.94** | **5479.02** |
| **71** | **BYM** | **RW2** | **TypeIV** | **No** | **5483.63** | **5479.86** |
| **72** | **iCAR** | **RW2** | **TypeII** | **Yes** | **5483.66** | **5480.04** |
| **73** | **BYM2** | **RW2** | **TypeIV** | **Yes** | **5483.75** | **5480.26** |
| **74** | **BYM** | **RW2** | **TypeIV** | **Yes** | **5483.77** | **5480.62** |
| **75** | **BYM2** | **RW2** | **TypeII** | **No** | **5483.78** | **5479.37** |
| **76** | **iCAR** | **RW2** | **TypeIV** | **Yes** | **5484.15** | **5481.25** |
| **77** | **Leroux** | **RW2** | **TypeIV** | **Yes** | **5484.61** | **5481.58** |
| **78** | **Leroux** | **RW2** | **TypeII** | **Yes** | **5484.74** | **5480.46** |
| **79** | **iCAR** | **RW2** | **TypeIV** | **No** | **5484.93** | **5482.04** |
| **80** | **BYM2** | **RW2** | **TypeIV** | **No** | **5490.55** | **5487.39** |
