# Supplementary material for: Spatio-temporal inequalities in vulvar cancer mortality in Spain, 1999–2023: a nationwide Bayesian analysis
Source: Clin Transl Oncol. 2026 May 25;28(11):5255–62. doi: 10.1007/s12094-026-04408-3 (PMC13694372; doi:10.1007/s12094-026-04408-3)
Supplement: Supplementary file 2 [file 12094_2026_4408_MOESM2_ESM.docx]

**Table S2. Province-level observed and model-based mortality estimates for vulvar cancer in Spain.**
Observed and expected number of deaths, posterior mean relative risk (RR), 95% credible intervals (CrI), and posterior probability of increased risk (PP(RR > 1)) for vulvar cancer mortality across 50 Spanish provinces and 2 autonomous cities. Expected counts are derived from the national standard population, and RR estimates are obtained from the final Bayesian spatio-temporal model.

| Province | Observed | Expected | RR | RR_li | RR_ls | PP |
| --- | --- | --- | --- | --- | --- | --- |
| Álava | 40 | 53.21 | 0.89 | 0.76 | 1.04 | 0.08 |
| Albacete | 70 | 64.37 | 0.99 | 0.84 | 1.16 | 0.43 |
| Alicante | 230 | 266.86 | 0.86 | 0.76 | 0.96 | 0.00 |
| Almería | 88 | 78.22 | 1.02 | 0.87 | 1.19 | 0.59 |
| Ávila | 49 | 39.73 | 1.05 | 0.90 | 1.24 | 0.73 |
| Badajoz | 160 | 114.33 | 1.25 | 1.10 | 1.43 | 1.00 |
| Balears (Illes) | 157 | 140.96 | 1.05 | 0.92 | 1.19 | 0.75 |
| Barcelona | 779 | 905.16 | 0.85 | 0.80 | 0.92 | 0.00 |
| Burgos | 79 | 77.44 | 0.98 | 0.85 | 1.13 | 0.39 |
| Cáceres | 90 | 81.91 | 1.06 | 0.90 | 1.23 | 0.75 |
| Cádiz | 198 | 149.77 | 1.21 | 1.07 | 1.36 | 1.00 |
| Castellón | 105 | 89.96 | 1.04 | 0.90 | 1.21 | 0.70 |
| Ciudad Real | 104 | 90.76 | 1.05 | 0.91 | 1.22 | 0.74 |
| Córdoba | 133 | 132.68 | 1.00 | 0.89 | 1.13 | 0.51 |
| Coruña (A) | 263 | 238.07 | 1.06 | 0.95 | 1.19 | 0.87 |
| Cuenca | 49 | 44.66 | 0.99 | 0.83 | 1.19 | 0.45 |
| Girona | 119 | 111.70 | 1.00 | 0.87 | 1.15 | 0.50 |
| Granada | 105 | 133.67 | 0.86 | 0.75 | 0.99 | 0.02 |
| Guadalajara | 33 | 36.83 | 0.92 | 0.76 | 1.12 | 0.20 |
| Guipúzcoa | 119 | 134.10 | 0.90 | 0.79 | 1.04 | 0.08 |
| Huelva | 98 | 70.62 | 1.20 | 1.04 | 1.39 | 0.99 |
| Huesca | 59 | 46.94 | 1.06 | 0.90 | 1.26 | 0.76 |
| Jaén | 107 | 107.84 | 0.97 | 0.84 | 1.12 | 0.34 |
| León | 120 | 120.00 | 0.98 | 0.85 | 1.13 | 0.38 |
| Lleida | 80 | 75.91 | 1.00 | 0.86 | 1.17 | 0.52 |
| Rioja (La) | 55 | 57.40 | 0.95 | 0.81 | 1.11 | 0.26 |
| Lugo | 124 | 95.47 | 1.16 | 1.00 | 1.34 | 0.98 |
| Madrid | 816 | 958.79 | 0.85 | 0.79 | 0.91 | 0.00 |
| Málaga | 217 | 204.40 | 1.03 | 0.92 | 1.16 | 0.72 |
| Murcia | 156 | 183.36 | 0.86 | 0.75 | 0.98 | 0.01 |
| Navarra | 114 | 110.21 | 0.98 | 0.86 | 1.13 | 0.41 |
| Ourense | 103 | 94.07 | 1.05 | 0.91 | 1.22 | 0.75 |
| Asturias | 236 | 242.49 | 0.96 | 0.86 | 1.07 | 0.25 |
| Palencia | 39 | 40.01 | 0.97 | 0.82 | 1.15 | 0.36 |
| Palmas (Las) | 120 | 114.44 | 1.03 | 0.89 | 1.18 | 0.64 |
| Pontevedra | 202 | 179.80 | 1.08 | 0.96 | 1.22 | 0.89 |
| Salamanca | 75 | 83.11 | 0.94 | 0.80 | 1.11 | 0.24 |
| Santa Cruz de Tenerife | 126 | 126.75 | 0.99 | 0.86 | 1.14 | 0.46 |
| Cantabria | 104 | 112.25 | 0.93 | 0.80 | 1.07 | 0.16 |
| Segovia | 42 | 34.04 | 1.04 | 0.88 | 1.24 | 0.67 |
| Sevilla | 315 | 253.62 | 1.18 | 1.07 | 1.31 | 1.00 |
| Soria | 23 | 23.46 | 0.96 | 0.79 | 1.16 | 0.32 |
| Tarragona | 120 | 119.32 | 0.98 | 0.86 | 1.13 | 0.41 |
| Teruel | 27 | 32.33 | 0.92 | 0.76 | 1.12 | 0.21 |
| Toledo | 122 | 106.73 | 1.07 | 0.93 | 1.23 | 0.82 |
| Valencia | 347 | 393.61 | 0.87 | 0.80 | 0.96 | 0.00 |
| Valladolid | 100 | 96.68 | 1.01 | 0.87 | 1.16 | 0.53 |
| Vizcaya | 256 | 226.67 | 1.05 | 0.94 | 1.17 | 0.82 |
| Zamora | 65 | 53.10 | 1.07 | 0.92 | 1.24 | 0.79 |
| Zaragoza | 190 | 180.32 | 1.01 | 0.90 | 1.14 | 0.57 |
| Ceuta | 9 | 7.71 | 1.05 | 0.87 | 1.26 | 0.68 |
| Melilla | 6 | 7.15 | 0.97 | 0.77 | 1.21 | 0.39 |
