# Supplementary material for: Spatio-temporal inequalities in vulvar cancer mortality in Spain, 1999–2023: a nationwide Bayesian analysis
Source: Clin Transl Oncol. 2026 May 25;28(11):5255–62. doi: 10.1007/s12094-026-04408-3 (PMC13694372; doi:10.1007/s12094-026-04408-3)
Supplement: Supplementary file 3 [file 12094_2026_4408_MOESM3_ESM.docx]

**STROBE Checklist**

*Spatio-Temporal Inequalities in Vulvar Cancer Mortality in Spain, 1999–2023: A Nationwide Bayesian Analysis*

| **#** | **Item** | **STROBE recommendation** | **Status** | **Location / notes** |
| --- | --- | --- | --- | --- |
| **Title & Abstract** |  |  |  |  |
| 1 | **Title & abstract** | Indicate study design; provide informative abstract | **Yes** | *Title states "nationwide Bayesian analysis"; abstract structured with Objectives/Methods/Results/Conclusions* |
| **Introduction** |  |  |  |  |
| 2 | **Background / rationale** | Explain scientific background and rationale for the study | **Yes** | *Introduction reviews global epidemiology, HPV pathways, and Spanish data gaps* |
| 3 | **Objectives** | State specific objectives, including pre-specified hypotheses | **Yes** | *Last paragraph of Introduction states aim explicitly* |
| **Methods** |  |  |  |  |
| 4 | **Study design** | Present key elements of study design early | **Yes** | *"Nationwide population-based ecological study" — Methods, first paragraph* |
| 5 | **Setting** | Describe setting, locations, and relevant dates | **Yes** | *Spain, 52 provinces, 1999–2023; data from INE* |
| 6 | **Participants** | Eligibility criteria; sources/methods of selection; for ecological: units of analysis and aggregation | **Yes** | *Ecological units are 52 Spanish provinces; ICD-10 C51; women only* |
| 7 | **Variables** | Define all outcomes, exposures, covariates; give diagnostic criteria | **Yes** | *Outcome (vulvar cancer mortality, ICD-10 C51) defined; no individual-level covariates — acknowledged as limitation* |
| 8 | **Data sources / measurement** | For each variable, give sources of data and details of assessment | **Yes** | *INE mortality and population data; indirect standardisation described* |
| 9 | **Bias** | Describe efforts to address potential sources of bias | **Yes** | *Ecological fallacy mentioned in Limitations; Bayesian smoothing noted for small-area uncertainty; no formal bias analysis* |
| 10 | **Study size** | Explain how study size was arrived at | **N/A** | *Whole-population study; no sample size calculation needed* |
| 11 | **Quantitative variables** | Explain handling of quantitative variables; describe groupings | **Yes** | *Five-year age groups; RR with 95% credible intervals; posterior probability thresholds stated (0.20, 0.80, 0.95)* |
| 12 | **Statistical methods** | Describe all statistical methods; explain how confounding was addressed; describe sensitivity analyses | **Yes** | *Poisson-INLA Bayesian spatio-temporal models; 4 spatial structures (iCAR, BYM, BYM2, Leroux); RW1/RW2; Knorr-Held Types I–IV; DIC/WAIC; 80 candidate models* |
| **Results** |  |  |  |  |
| 13 | **Participants** | Report numbers at each stage; give reasons for non-participation | **N/A** | *Ecological study; all 52 geographical units included throughout* |
| 14 | **Descriptive data** | Give characteristics of study participants; indicate missing data | **Yes** | *Results – national trend and spatial variability* |
| 15 | **Outcome data** | Report numbers of outcome events or summary measures over time | **Yes** | *RR and CrI reported for high/low-risk provinces* |
| 16 | **Main results** | Give unadjusted and adjusted estimates; report precision; describe confounders | **Yes** | *Smoothed RR with 95% CrI and posterior probabilities; spatial/temporal/interaction variance decomposition (68.8%, 8.2%, 23.0%)* |
| 17 | **Other analyses** | Report other analyses done — subgroups, interactions, sensitivity | **Yes** | *80 models compared across spatial structures and temporal terms; robustness confirmed across iCAR and BYM2 alternatives* |
| **Discussion** |  |  |  |  |
| 18 | **Key results** | Summarise key results with reference to study objectives | **Yes** | *Three principal findings explicitly listed at start of Discussion* |
| 19 | **Limitations** | Discuss limitations, sources of bias, and direction of potential bias | **Yes** | *Dedicated limitations paragraph: ecological fallacy, no stage/HPV/socioeconomic data, residual uncertainty in small areas, mortality vs incidence* |
| 20 | **Interpretation** | Cautious overall interpretation; discuss findings in context of evidence | **Yes** | *Results contextualised against England, Sweden, Germany; multiple mechanisms discussed; vaccination implications noted* |
| 21 | **Generalisability** | Discuss generalisability (external validity) | **Yes** | *Results compared to European data; Spain-specific context emphasised* |
| **Other Information** |  |  |  |  |
| 22 | **Funding** | Give funding source and role of funders | **Yes** | *Declarations section: no specific funding received* |

**Status key:** Yes = fully reported	N/A = not applicable to this study design
